# A Text-Book of Insanity and Other Mental Disorders

**Published:** 1914-12

**Authors:** 


**A Text-Book of Insanity and other Mental Disorders.** By Charles Arthur Mercier, M.D. Second Edition. Pp. viii. 348. London: George Allen and Unwin, Ltd. 1914.

If Dr. Mercier is critical of the work of others, he is punctilious in the preparation of his own; accordingly, he is careful to previse the purposes and limitations of this book, so that within these only is criticism appropriate. It is set out for the student and practitioner (and fulfils this intent far better than most manuals so prefaced), but it is also designed to profit the alienist, whom one might suppose from sundry observations is still rather benighted amongst the mists of a vague psychology. We consider this book quite an ideal one for the student, and a valuable one for the alienist. There has been a long series of text-books issued on insanity, some excellent in their kind, some redeemed by individual researches, others which in the words of scripture had better have never been born. Those issued specially for the student, according to the scheme of the publisher, have mostly been treated in stereotyped manner; for this examiners have been largely responsible. Each succeeding work seeks to cover a larger ground; there is necessarily inadequate, and in relation to the beginner, unprofitable, summary of anatomy, physiology, pathology, experimental and other psychology. Most of this, as Dr. Mercier indicates, is superfluous under the circumstances. He has therefore discarded the majority of these encumbrances, and has adopted and elaborated a scheme of which the germ is to be found in the older treatises, that is the constitution of mind in its various faculties; an eminently practical mode of regard. These faculties have, however, for the first time been sifted, grouped, and stratified in their order of evolution, and the result is a singularly clear exposition of normal mind which the student can at once grasp, and of whose parts the relationships can be estimated. This plan having been established, disordered states can be arranged in an equally lucid manner; and here one may comment on the ingenious way in which the clinical aspect of insanity has been treated *pari passu* with morbid psychology, so that its general features are elucidated before a single chapter has been devoted to any form or kind of mental disorder. The section on "Classification" is beyond mere theoretical importance, dealing as it does with the distinctions between primary and derived insanity, which must be seized in forming estimate of any case.

Pathology is not treated to any extent. This we think is wise. The description of the morbid anatomy of general paralysis is, however, excellent. Other sections of the book deal with lunacy law and criminal responsibility.

Dr. Mercier's definition of paranoia appears to correspond to hallucinatory delirium so-called, whilst the ordinary significance, we believe, answers to the "délire d'interpretation." To this latter Mercier's delirium of exaltation or abasement seemingly corresponds. This is the second edition, and should be by no means the last. Let us, in conclusion, express our gratitude to Dr. Mercier that he has excluded that veritable "King Charles's head" of modern text-books, dementia precox, as also manic-depressive insanity!

